# The impact of tau-PET in a selected memory clinic cohort: rationale and design of the TAP-TAU study

**DOI:** 10.1186/s13195-024-01588-4

**Published:** 2024-10-19

**Authors:** Marie R. Vermeiren, Joost Somsen, Gert Luurtsema, Fransje E. Reesink, Nicolaas A. Verwey, Liesbeth Hempenius, Nelleke Tolboom, Geert Jan Biessels, J. Matthijs Biesbroek, Meike W. Vernooij, Sophie E. M. Veldhuijzen van Zanten, Harro Seelaar, Emma M. Coomans, Charlotte E. Teunissen, Afina W. Lemstra, Argonde C. van Harten, Leonie N. C. Visser, Wiesje M. van der Flier, Elsmarieke van de Giessen, Rik Ossenkoppele

**Affiliations:** 1 Alzheimer Center Amsterdam, Amsterdam UMC, Vrije Universiteit, Amsterdam, Netherlands; 2 Department of Radiology & Nuclear Medicine, Amsterdam UMC, Vrije Universiteit, Amsterdam, Netherlands; 3 Amsterdam Neuroscience, Brain Imaging, Amsterdam, Netherlands; 4 Department of Nuclear Medicine and Molecular Imaging, University of Groningen, University Medical Center Groningen, Groningen, Netherlands; 5 Department of Neurology, University of Groningen, University Medical Center Groningen, Groningen, Netherlands; 6 Department of Neurology, Medical Center Leeuwarden, Leeuwarden, Netherlands; 7 Amsterdam Neuroscience, Neurodegeneration, Amsterdam, Netherlands; 8 Geriatric Center, Medical Center Leeuwarden, Leeuwarden, Netherlands; 9 Department of Radiology and Nuclear Medicine, University Medical Centre Utrecht, Utrecht, Netherlands; 10 Department of Neurology and Neurosurgery, UMC Utrecht Brain Center, University Medical Center Utrecht, Utrecht, Netherlands; 11 Department of Neurology, Diakonessenhuis Hospital, Utrecht, Netherlands; 12 Department of Radiology and Nuclear Medicine, Erasmus University Medical Center, Rotterdam, Netherlands; 13 Department of Epidemiology, Erasmus University Medical Center, Rotterdam, Netherlands; 14 Department of Neurology, Erasmus University Medical Center, Rotterdam, Netherlands; 15 Neurochemistry Laboratory, Amsterdam UMC, Amsterdam, Netherlands; 16 Division of Clinical Geriatrics, Center for Alzheimer Research, Department of Neurobiology, Care Sciences and Society, Karolinska Institutet, Stockholm, Sweden; 17 Department of Medical Psychology, Amsterdam UMC, University of Amsterdam, Amsterdam, Netherlands; 18 Epidemiology and Data Science, Amsterdam UMC, Amsterdam, Netherlands; 19 Clinical Memory Research Unit, Lund University, Lund, Sweden

**Keywords:** Alzheimer’s disease, Dementia, Tau, Positron emission tomography, Prospective studies

## Abstract

**Background:**

Tau-PET is a diagnostic tool with high sensitivity and specificity for discriminating Alzheimer’s disease (AD) dementia from other neurodegenerative disorders in well-controlled research environments. The role of tau-PET in real-world clinical practice, however, remains to be established. The aim of the TAP-TAU study is therefore to investigate the impact of tau-PET in clinical practice.

**Methods:**

TAP-TAU is a prospective, longitudinal multi-center study in 300 patients (≥ 50 years old) with mild cognitive impairment or mild dementia across five Dutch memory clinics. Patients are eligible if diagnostic certainty is < 85% after routine dementia screening and if the differential diagnosis includes AD. More specifically, we will include patients who (i) are suspected of having mixed pathology (e.g., AD and vascular pathology), (ii) have an atypical clinical presentation, and/or (iii) show conflicting or inconclusive outcomes on other tests (e.g., magnetic resonance imaging or cerebrospinal fluid). Participants will undergo a [^18^F]flortaucipir tau-PET scan, blood-based biomarker sampling, and fill out questionnaires on patient reported outcomes and experiences. The primary outcomes are change (pre- versus post- tau-PET) in diagnosis, diagnostic certainty, patient management and patient anxiety and uncertainty. Secondary outcome measures are head-to-head comparisons between tau-PET and less invasive and lower cost diagnostic tools such as novel blood-based biomarkers and artificial intelligence-based classifiers.

**Results:**

TAP-TAU has been approved by the Medical Ethics Committee of the Amsterdam UMC. The first participant is expected to be included in October 2024.

**Conclusions:**

In TAP-TAU, we will investigate the added clinical value of tau-PET in a real-world clinical setting, including memory clinic patients with diagnostic uncertainty after routine work-up. Findings of our study may contribute to recommendations regarding which patients would benefit most from assessment with tau-PET. This study is timely in the dawning era of disease modifying treatments as an accurate etiological diagnosis becomes increasingly important.

**Trial registration:**

This trial is registered and authorized on December 21st, 2023 in EU Clinical Trials with registration number 2023-505430-10-00.

**Supplementary Information:**

The online version contains supplementary material available at 10.1186/s13195-024-01588-4.

## Background

Alzheimer’s disease (AD) is pathologically characterized by cerebral amyloid-β (Aβ) plaques and tau neurofibrillary tangles (NFTs) [1]. The differential diagnosis of AD versus other neurological and psychiatric disorders can be challenging due to substantial overlap in clinical symptoms, atypical presentations and patterns of neurodegeneration identified with magnetic resonance imaging (MRI) and [^18^F]-2fluoro-2deoxydglucose positron emission tomography ([^18^F]FDG-PET) [1–3]. Moreover, as indicated by post-mortem data, the majority of individuals with pathologically defined AD have concomitant pathologies, in particular vascular, Lewy body and TAR DNA-binding protein 43 (TDP-43) pathologies [4–9]. In view of the forthcoming implementation of disease modifying treatments (DMTs) for AD, it becomes increasingly important to accurately determine the primary cause of cognitive symptoms using disease-specific biomarkers.

The utility of MRI and [^18^F]FDG-PET are limited as they lack molecular specificity to detect the neuropathology that underlies the observed pattern of neurodegeneration and hypometabolism. Although the sensitivity of Aβ biomarkers of AD pathology, measured in cerebral spinal fluid (CSF) and with PET, are excellent, their specificity for differentiating symptomatic AD from other neurodegenerative disorders is modest [10, 11]. Biomarkers of cerebral Aβ pathology become abnormal up to 20 years before clinical symptoms occur [12, 13]. Therefore, incidental Aβ pathology is common, especially at an older age [11, 14–19]. Moreover, elevated concentrations of phosphorylated tau (p-tau) in CSF and plasma reflect early alterations in tau metabolism and are commonly observed with advancing age [14, 17, 18, 20]. Consequently, in memory clinic patients with abnormal Aβ and p-tau biomarkers it may not be clear whether AD pathology or another neurodegenerative disease is the primary etiology underlying the cognitive deficits.

In contrast, tau positron emission tomography (tau-PET) informs on the likelihood that AD pathology is the driver of symptoms in cognitively impaired individuals [21]. Common tau-PET tracers, such as [^18^F]flortaucipir, bind specifically to the combination of 3R/4R isoforms of tau protein that are characteristic of AD [22–26]. Tau-PET shows excellent diagnostic performance in distinguishing AD versus other neurodegenerative disorders [27–30]. In comparison to other currently available biomarkers, the specificity (~ 90%) of tau-PET is superior [27, 28, 30, 31]. Extensive literature provides evidence that tau-PET is strongly associated with cognition, clinical symptoms and neurodegeneration [32–37]. A positive tau-PET scan in a cognitively impaired patient strongly increases the likelihood that AD is the primary etiology underlying the clinical syndrome. While Aβ and p-tau biomarkers are useful to exclude a diagnosis of AD, tau-PET can be used both to rule in and rule out a diagnosis of AD [38]. Consequently, the U.S. Food and Drug Administration (FDA) has approved [^18^F]flortaucipir as a tau-PET tracer for clinical use in patients with cognitive impairment who are being evaluated for AD [22].

The additional value of tau-PET in real-world clinical practice, however, remains to be established [39]. We hypothesize that tau-PET will incur changes of the pre-PET clinical diagnosis and patient management and will improve diagnostic certainty and patient-centered outcomes. Since tau-PET is a relatively expensive and not widely available technique, it will likely be used selectively in future clinical practice. Therefore, the primary aim of TAP-TAU is to investigate the impact of tau-PET in a memory clinic population in the MCI and mild dementia stadium with diagnostic uncertainty after routine work-up. The secondary objective is a head-to-head comparison between tau-PET and less-invasive and cheaper diagnostic tools such as novel blood-based biomarkers and artificial intelligence (AI) based classifiers.

## Methods

### Study design

TAP-TAU is a prospective, longitudinal, multi-center study in cognitively impaired patients who visit the memory clinic and have significant diagnostic uncertainty. The study is carried out in memory clinics of four academic medical centers and one non-academic medical center in the Netherlands; Amsterdam University Medical Center, Erasmus Medical Center Rotterdam, University Medical Center Utrecht, University Medical Center Groningen and Medical Center Leeuwarden. TAP-TAU is embedded in the TAP-Dementia Consortium, where T stands for timely, A for accurate and P for personalized. The TAP-Dementia Consortium aims to improve the diagnostic process in AD (www.tap-dementia.nl).

**Fig. 1:**
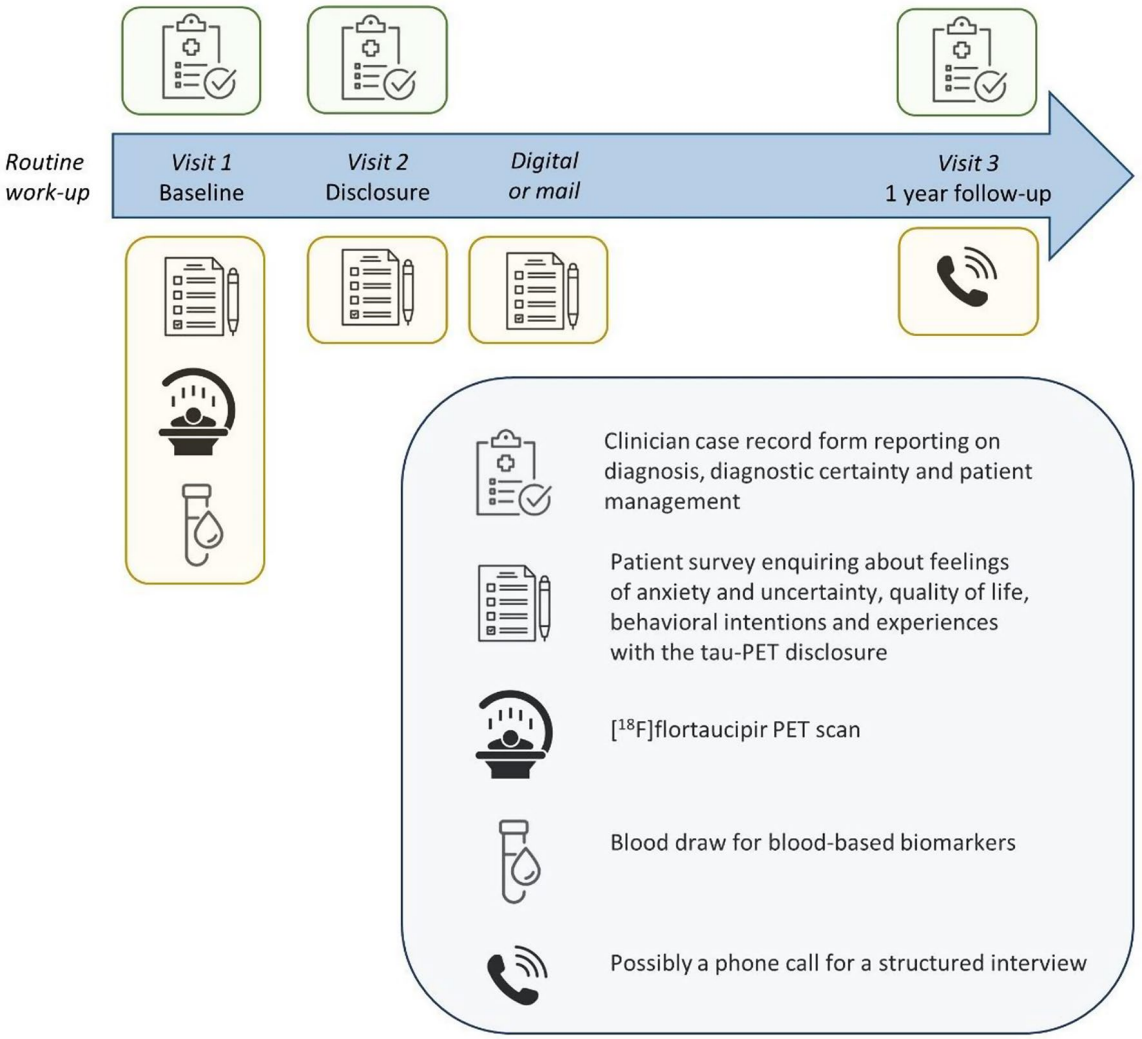
TAP-TAU study timeline and procedures

At baseline, participants undergo a tau-PET scan and a blood draw for the assessment of blood-based biomarkers and they complete a questionnaire on patient reported outcomes (PROs) and experiences (PREs). The attending physician completes a baseline case record form (CRF) on diagnosis, diagnostic confidence and patient management (see Supplementary Materials for the translated clinician CRF). The CRF is completed again after the physician receives the tau-PET result. A few weeks after the tau-PET scan, participants have a visit with their physician for the disclosure of the tau-PET biomarker result. This is directly followed by the completion of a questionnaire on PROs and PREs, which is repeated again several weeks later either digitally or by mail. One year after the tau-PET scan, participants are re-evaluated clinically by their physician and the CRF is once again completed to verify whether the disease manifestation is compatible with their post-PET diagnosis (surrogate gold standard). A selection of 20 participants with varying experiences will be invited to a telephone interview after one year, using a semi-structured topic guide assessing the effect of the tau-PET scan on the participant’s life. A schematic overview of the TAP-TAU study design and procedures is provided in Fig. 1.

### Participants

**Fig. 2:**
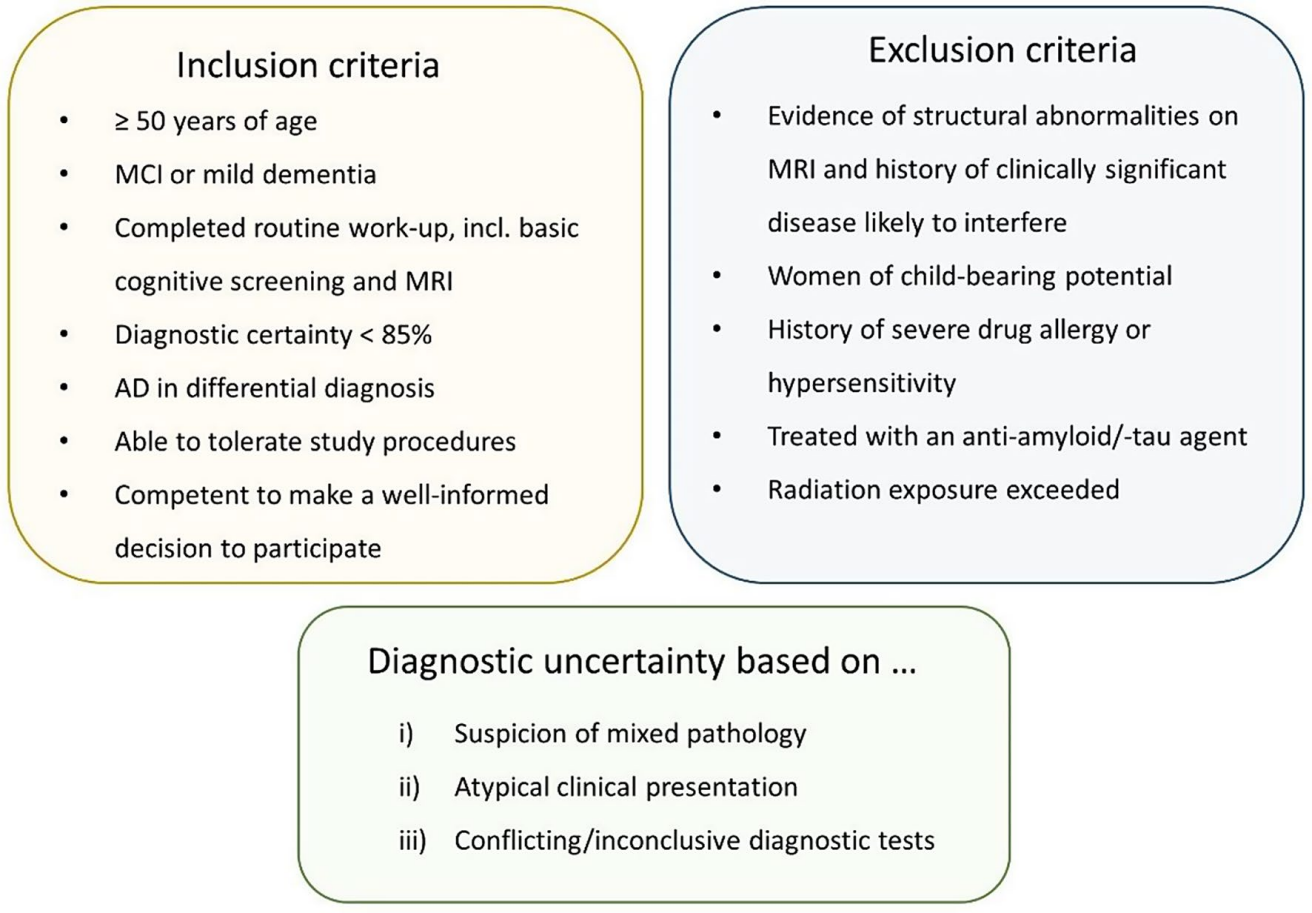
Criteria for participation in the TAP-TAU study. MCI, mild cognitive impairment; MRI, magnetic resonance imaging; AD, Alzheimer’s disease

We aim to include 300 participants in the tau-PET study. Participants are recruited from specialized memory clinics at Amsterdam University Medical Center (*n* = 150), Erasmus Medical Center (*n* = 50), University Medical Center Utrecht and Diakonessenhuis (participants from Diakonessenhuis are referred to Utrecht for the study, together *n* = 50), University Medical Center Groningen and Medical Center Leeuwarden (together *n* = 50). An overview of inclusion and exclusion criteria is given in Fig. 2. In addition, patients at Amsterdam University Medical Center who meet eligibility criteria but do not wish to participate in the PET study, are asked to take part in the control group (*n* = 60). From controls only data from electronic medical files is collected and their physician fills out the CRF at baseline and after one year.

#### Inclusion criteria

Patients are eligible if they are 50 years of age or above and are clinically in the MCI (mild cognitive impairment, CDR 0.5) or mild dementia stage (CDR 1). Before inclusion, participants should have completed regular diagnostic trajectories, which differ by recruitment site but at least includes basic cognitive screening tests and MRI scanning with a 3D T1-weighted sequence. Patients are eligible if after the routine work-up substantial diagnostic uncertainty in the aetiology remains according to the treating physician and AD is in the differential diagnosis. Confidence < 85% on a visual analogue scale ranging from 0 to 100% is considered as substantial diagnostic uncertainty based on findings from prospective PET studies similar in design [40–42]). More specifically, this includes individuals with.

i) suspicion of mixed pathology,
ii) presence of an atypical clinical presentation, and/or
iii) conflicting/inconclusive information from other diagnostic tests like MRI or cerebral spinal fluid (CSF).

Participants should be able to tolerate study procedures and be competent to make a well-informed decision to participate in this study based on the expert opinion of the attending physician.

#### Exclusion criteria

Exclusion criteria are evidence of structural abnormalities such as major stroke or mass on MRI and a history of any clinically significant medical condition likely to interfere with the clinical presentation and/or interpretation of the PET scan, as determined by the principal investigator. Participants are excluded from the tau-PET group if they are women of child-bearing potential, have a relevant history of severe drug allergy or hypersensitivity, have ever been treated with an anti-amyloid drug or tau agent and if they have been injected with a previously administered radiopharmaceutical within 6 terminal half-lives or when total yearly radiation exposure for research exceeds 11.3 mSv for females and 15.3 mSv for males.

### Study timeline and procedures

#### Screening and informed consent

Patients are asked if they are interested to participate in the TAP-TAU study by their attending physician. This generally takes place at the visit during which the results of routine diagnostic tests and possibly outcomes of multidisciplinary meetings are disclosed. An information letter about the study is provided. Participation in TAP-TAU can also be considered during follow-up when diagnostic uncertainty persists or arises following ancillary investigations or evolution in the patient’s clinical presentation. Patients are contacted after one week to inquire whether they are still interested to take part in the study. In- and exclusion criteria are checked and visits planned accordingly. Participants sign informed consent during the baseline visit in the presence of the study coordinator before any study procedures are performed. Baseline medical data from electronic medical files are collected and the attending physician completes the baseline CRF on diagnosis, diagnostic confidence and patient management.

#### [^18^F]flortaucipir PET procedures, analyses and rating

[^18^F]flortaucipir PET scans are performed during the baseline visit on large field of view PET/CT scanners (Amsterdam University Medical Center, University Medical Center Groningen) or conventional PET/CT-scanners (University Medical Center Utrecht, Erasmus Medical Center Rotterdam). Tau-PET scans for participants at Medical Center Leeuwarden are performed at the University Medical Center Groningen. For quantification purposes scans from different scanners are harmonized using a Hoffman phantom scan [43]. Before scanning, an intravenous cannula is inserted for infusion of the tracer dose. Subjects receive a single intravenous bolus of approximately 140 MBq of [^18^F]flortaucipir (for large field of view PET/CT-scanners) or 225 MBq of [^18^F]flortaucipir (for conventional PET/CT-scanners). During scanning, the head is immobilized to reduce movement artefacts and, using laser beams, positioned within the centre of axial and transaxial fields of view, such that the orbito-meatal line is parallel to the detectors. All PET scans are taken under standard resting conditions. Scan duration of [^18^F]flortaucipir in all patients is 80–100 min post-injection. Prior to the PET scan, a low-dose CT scan is performed for attenuation correction. Subjects will be observed continuously for signs of (serious) adverse events.

**Fig. 3:**
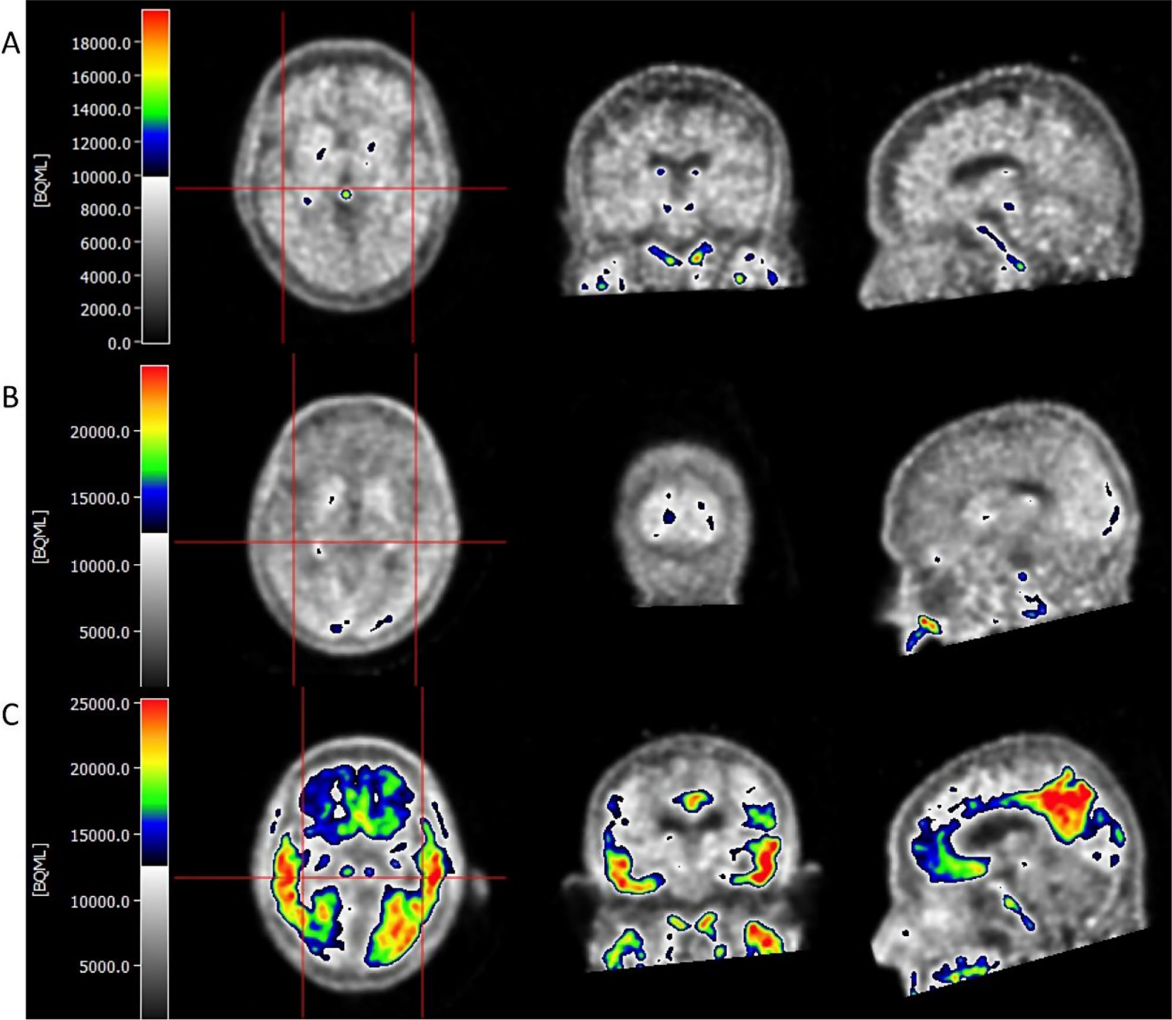
Example PET scans of a visually negative (**A**), moderately positive (**B**) and advanced positive (**C**) tau-PET scan determined following the FDA approved visual read method for [^18^F]flortaucipir. The scans are from study participants at the Amsterdam University Medical Center who participated in previous tau-PET studies

During reconstruction all corrections (e.g. attenuation, scatter, dead time, and normalisation) are applied. Images are prepared and visually read following the FDA approved metric for [^18^F]flortaucipir that used autopsy-confirmed tau load as gold standard [22]. A negative AD tau pattern is characterized by either no increase in neocortical activity or increased activity restricted to the mesial temporal, anterolateral temporal, and/or frontal areas. A moderate AD tau pattern exhibites increased neocortical activity in the posterolateral temporal or occipital regions. An advanced AD tau pattern is defined by increased neocortical activity in the parietal or precuneus regions, or by increased activity in the frontal region alongside increased activity in the posterolateral temporal, parietal, or occipital regions [22]. Example PET images of a negative, moderately positive and advanced positive tau-PET scan are shown in Fig. 3. This method has shown strong concordance with a quantitative measure for tau burden, the standardized uptake value ratio (SUVr) [44]. All PET scans are read centrally at the Amsterdam University Medical Center by two experienced nuclear medicine physicians blinded from clinical information (EvdG and NT) [22]. Visual ratings of the tau-PET scans, including a confidence score (ranging from 0 to 5) and a description of affected regions, are communicated to the treating physician in each clinical site. To verify the visual reads retrospectively, tau load is quantified using SUVr using whole cerebellar gray matter as a reference region in relevant regions-of-interest (ROI), e.g. the commonly used temporal meta-ROI [27] and a temporoparietal ROI (corresponding to regions that contribute to a positive visual read) [44].

#### Blood sampling

A blood draw is done prior to the PET scan via the venous cannula that is already placed for PET tracer injection. If no blood can be drawn from the cannula due to practical or logistic reasons, a venepuncture is performed. Two 6 mL EDTA tubes are collected under non-fasting conditions. Within 2 h of collection, plasma is centrifuged for 10 min at 1800xg at room temperature and the plasma is stored at -80 degrees Celsius in aliquots of 500 microL in prolyproylene cryovials until further analysis. Measurements of blood-based biomarkers specific to dementia aetiologies, such as aβ42/40, (p-)tau isoforms, GFAP, NfL, ApoE genotype and others are performed centrally at the Neurochemistry lab of Amsterdam University Medical Center. Among others the Alzheimer blood test from Roche is used.

#### Disclosure

Following the receival of the tau-PET result, physicians re-asses diagnosis, diagnostic certainty and patient management utilizing the clinician CRF. Participants have a visit with their physician after their PET scan during which the tau-PET results are disclosed. This is immediately followed by the completion of a questionnaire on PROs and PREs (see below) by the participant, assisted by a researcher and possibly a caregiver, to measure possible direct effects of the tau-PET disclosure. Approximately 4 weeks after the disclosure, when participants had time to reflect on the disclosure and their results, they again complete a questionnaire on PROs and PREs through a digital form or letter. Participants can choose not to know their tau-PET result.

#### Patient surveys

Participants complete questionnaires on PROs and PREs at three time points: at baseline, directly after disclosure of the tau-PET result and again approximately 4 weeks after disclosure. The patient questionnaire at baseline assesses medical knowledge, information need, motivation for the tau-PET, and behavior (e.g. intention to eat healthier). Moreover, validated questionnaires administered at baseline include the short version of the State-Trait Anxiety Inventory (STAI-S) [45, 46], measuring feelings of anxiety, selected items of the Mishel Uncertainty of Illness Scale (MUIS) [47], measuring feelings of uncertainty, and the ICEpop CAPability measure for Older people (ICECAP-O) [48], measuring quality of life. In both the questionnaire directly after the tau-PET disclosure and the questionnaire 4 weeks later, satisfaction with the received information is measured utilizing a selection of items from the EORTC Quality of Life Questionnaire-Information module [49]. Moreover, measurements of anxiety, uncertainty and quality of life (STAI-S, MUIS, ICECAP-O) are repeated at both time points post-disclosure. The patient survey directly after the disclosure also includes questions testing the information recall and satisfaction with the disclosure conversation, using the short form of the Patient Satisfaction Questionnaire [49]. In the last survey, we additionally measure regret, using selected items of the Decision Regret Scale [50], complemented by self-formulated questions on experiences with the tau-PET scan, and behavior relating to health. Of all validated questionnaires translated versions in Dutch are used. A year after disclosure, we invite a subset of patients to participate in a structured interview to qualitatively assess the effects of the tau-PET scan on their (quality of) life.

#### Follow-up

Participants undergo clinical follow-up after one year with a visit to their attending physician. This visit includes a short cognitive test battery (incl. MMSE and/or MoCA) to verify the diagnosis and assess clinical progression. Physicians then once again fill out the CRF on diagnosis, diagnostic certainty and patient management. Because the control group participants are all patients at the Amsterdam University Medical Center, a follow-up visit at one year is already part of standard care.

### Outcomes

The primary endpoints are pre- versus post- tau-PET change in diagnosis, confidence of the clinician in the diagnosis, patient management and patient anxiety and uncertainty. The pre-PET and post-PET diagnosis, diagnostic certainty and patient management are described by the attending physician in detail using the clinician CRF (included in Supplementary Materials). In general, diagnostic categories are AD, non-AD with specification of suspected underlying pathology, mixed pathology (AD and non-AD), which are further specified. Diagnostic confidence is measured on a continuous visual analogue scale ranging from 0 to 100%. Patient management includes increase or reduction in ancillary investigations, initiation or withdrawal of medication and initiation or withdrawal of care. We also assess whether tau-PET influences hypothetical prescription of DMTs for AD. Endpoints of anxiety and uncertainty are described in “Study timeline and procedures: Patient surveys”. Change in diagnosis, diagnostic confidence and management are compared to the control group and between subgroups, as described in “Statistical analysis plan”. The secondary objective is to compare tau-PET to (combinations of) less expensive and more accessible diagnostic tools. To this end, performance of tau-PET is compared against novel blood-based biomarkers and artificial intelligence based classifiers that will incorporate available clinical information (e.g. neuropsychological test scores, MRI, CSF). The development of the AI classification model is currently ongoing within the TAP-DANCE project, part of the TAP-Dementia consortium.

### Statistical analysis plan

For the primary objective, change in diagnosis and patient management is assessed as percentage for the tau-PET group, control group, per patient subgroup (i.e., (i) suspicion of mixed pathology, (ii) presence of an atypical clinical presentation, and/or (iii) conflicting/inconclusive information from other diagnostic tests), stratified by availability of amyloid biomarkers and stratified by tau-PET visual read result. Pre- and post-PET differences in diagnosis and patient management are analyzed using the McNemar test. For comparisons between groups the chi-square test is utilized. Differences in continuous measures of diagnostic confidence and patient wellbeing within groups and between the aforementioned groups are assessed using ANOVA. For the secondary objective, the overall concordance rate between tau-PET and blood-based biomarkers is calculated as a percentage of concordant patients. We investigate the potential interchangeability between plasma biomarkers and tau-PET and the efficiency of a sequential approach. Based on the statistical parameters obtained in the first part, we examine whether performance of plasma biomarker testing first can reduce the number of tau-PET scans needed. The output of the AI model to AD vs. non-AD is dichotomized and the overall concordance rate between tau-PET and the AI model is calculated as a percentage of concordant patients. To further validate the concordance rates, we perform receiver operating characteristic analysis to calculate the area under the curve of plasma biomarker and AI-positivity ratio for tau-PET positivity. Results from the structured telephone interviews will be analyzed using qualitative content analysis.

### Ethical consideration and data sharing

TAP-TAU is conducted in accordance with the Medical Research Involving Human Subjects Act (WMO) and according to the principles of the World Medical Association (WMA) Declaration of Helsinki. The Medical Ethics Committee from the Amsterdam University Medical Center has approved the study (EU clinical trials number 2023-505430-10-00). The handling of data is in agreement with the EU General Data Protection Regulation and the Dutch Act on Implementation of the General Data Protection Regulation. Participant’s privacy and confidentiality is respected throughout the study.

## Results

On December 21st, 2023 the Medical Ethics Committee of the Amsterdam University Medical Center approved the TAP-TAU study. The first participant is expected to be included in October 2024.

## Discussion

The TAP-TAU study aims to investigate the added clinical value of tau-PET on top of routine work-up diagnostics in a group of memory clinic patients with diagnostic uncertainty, and consequently to identify which patients benefit most from a tau-PET scan. We will compare the performance of tau-PET to more affordable and scalable biomarkers and investigate whether and how they can be used in combination. We envision that our findings will provide evidence from clinical practice to support appropriate use criteria for tau-PET and ultimately assist clinicians, patients and caregivers in differential diagnosis procedures.

This study is timely in the dawning era of DMTs as vigilance concerning mixed pathologies and atypical presentations in the memory clinic population grows. There is an urging need for rapid and accurate identification of underlying neurodegenerative pathologies at play in memory clinic patients. Moreover, it will be relevant to gain insight into the proportion of considerable NFT burden in patients without a typical AD presentation and presence of co-pathologies.

Evidence is accumulating that application of AD biomarkers leads to improved diagnostic accuracy, increased diagnostic confidence of clinicians, and frequent changes in patient management, even in the absence of DMTs [40, 42, 51–55]. Large prospective diagnostic studies show that revealing amyloid-PET results led to a change in diagnosis in 23–44% of cases [40, 42, 51, 52, 54]. Moreover, amyloid-PET prompts a change in patient management in 24–65% of cases, ranging from drug therapy to ancillary tests and future planning [40, 54]. A study using data provided by the Dutch government and insurance companies provides evidence that an accurate diagnosis by means of amyloid-PET contributes to delayed institutionalization, lower mortality and a reduction in care costs [55]. By virtue of its higher accuracy for the diagnosis of symptomatic AD compared to amyloid-PET [11, 16, 27, 28, 31], we expect that the impact of tau-PET in clinical practice will be similar or even higher than that of amyloid-PET.

To date, studies examining the impact of tau-PET in clinical practice are scarce [51, 53, 56]. Two studies assessed the clinical value of tau-PET in a consecutive series of memory clinic patients without targeting specific patient groups [51, 53], showing that a change in diagnosis occurred after tau-PET in 28% of the participants when amyloid status was unknown and ~ 8% when amyloid status was available. It remains unclear though which patients would benefit most from tau-PET. As tau-PET has shown high performance in discriminating AD from non-AD neurodegenerative diseases, it is expected to have added value over current diagnostic processes in patients with residual diagnostic uncertainty after routine diagnostic work-up. However, due to decreased sensitivity of tau-PET at prodromal stages, when the extent of tau-PET uptake is lower, its value in early clinical stages is unclear [24, 27, 57]. TAP-TAU addresses some remaining gaps necessary for full maturity of tau-PET according to the strategic biomarker roadmap for the validation of Alzheimer’s diagnostic biomarkers. Quantifying the benefit of tau-PET in a real-world clinical context, by supporting the clinical diagnosis for patients within a research framework, TAP-TAU covers the aims of Phase 4. Additionally, our secondary objectives of comparing and combining biomarkers contribute to the aims of Phase 3 [39, 58].

As a nation-wide study, TAP-TAU strives for collaboration. Within the TAP-Dementia consortium, efforts from the TAP-TAU and TAP-PAT projects are combined to support implementation and communication of dementia biomarkers and harmonize the clinical work-up. Moreover, TAP-TAU works together with the TAP-DANCE and TAP-VaMP projects in the development of novel dementia biomarkers, such as AI based classifiers and blood-based biomarkers. Moreover, TAP-TAU will profit from the structure and network offered by the Dutch ABOARD cohort (https://www.alzheimer-europe.org/our-work/current-work/aboard?language_content_entity=en).

## Limitations

The non-randomized design of the TAP-TAU study can be considered as a limitation. After thorough deliberation we have opted to include a non-randomized control group instead. We argue that for our study goal the added value of randomization is not assured and instead could result in lower power. We acknowledge the potential bias that lies in the recruitment strategy for the control group. Possibly, patients who refuse a tau-PET scan and consent to be included in the control group are less uncertain and/or anxious about their diagnosis or perhaps their treating physician gave them less reason to feel so. We will take this potential bias into account when interpreting the results. We only collect control data at the Amsterdam University Medical Center as this is more feasible in the Amsterdam University Medical Center than in the other centers. However, bias due to this is limited since all centers but one are academic memory clinics and since the highest number of tau-PET scans will be acquired in Amsterdam. Due to logistic limitations of our study design, we will not compare the performance of tau-PET with the gold standard of post-mortem pathology. Instead, we will assess the clinical progression and diagnosis after one year as a surrogate gold standard, in line with previous clinical value studies [40, 51, 53, 54]. Another potential limitation of this study can be the heterogeneity of employed routine diagnostics in the various clinical sites, as e.g. some sites perform lumbar punctures more commonly than others. This can also be considered a strength as it more closely resembles clinical practice. Lastly, diagnostic confidence can vary between clinicians, which will mainly impact the baseline score as we primarily focus on the change in confidence.

## Conclusion

In TAP-TAU we will investigate the added clinical value of tau-PET in a selected memory clinic population with diagnostic uncertainty after routine work-up. Findings from this study may guide appropriate use criteria for tau-PET [59] and ultimately benefit patients and their caregivers. This study is timely in the dawning era of DMTs as vigilance concerning mixed pathologies and atypical presentations in the memory clinic population grows.

## Electronic supplementary material

Below is the link to the electronic supplementary material.

Supplementary Material 1

## Data Availability

No datasets were generated or analysed during the current study.

## Abbreviations

AD: Alzheimer’s disease
AI: Artificial intelligence
ANOVA: Analysis of Variance
ApoE: Apolipoproteïne E
Aβ: Amyloid-β
CDR: Clinical Dementia Rating scale
CRF: Case record form
CSF: Cerebral spinal fluid
CT: Computed tomography
DMT: Disease modifying therapy
EDTA: Ethylenediaminetetraacetic acid
FDA: U.S. Food and Drug Administration
FDG: Fluorodeoxyglucose
GFAP: Glial fibrillary acidic protein
ICECAP-O: ICEpop CAPability measure for Older people
MCI: Mild cognitive impairment
MMSE: Minimental state examination
MoCA: Montreal Cognitive Assessment
MRI: Magnetic resonance imaging
MUIS: Mishel Uncertainty of Illness Scale
NfL: Neurofilament light chain
p-tau: Phosphorylated tau
ROI: Region of interest
STAI-S: State-Trait Anxiety Inventory
SUVR: Standardized uptake value ratio
tau-PET: Tau positron emission tomography
TDP-43: TAR DNA-binding protein 43
WMA: World Medical Association
WMO: Medical Research Involving Human Subjects Act
