# Supplementary material for: The impact of tau-PET in a selected memory clinic cohort: rationale and design of the TAP-TAU study
Source: Alzheimers Res Ther. 2024 Oct 19;16:230. doi: 10.1186/s13195-024-01588-4 (PMC11490118; doi:10.1186/s13195-024-01588-4)
Supplement: Supplementary file 1 — Supplementary Material 1 [file 13195_2024_1588_MOESM1_ESM.docx]

**Clinician CRF TAP-TAU Study**

Study Number TAP-TAU

Year of Birth:

**MDO**

Date CRF completed:

Supervisor / treating physician (must be the same physician at all three time points):

BEFORE TAU PET

1. What is the clinical (syndrome) diagnosis? (choose one)

MCI (CDR = 0.5)

Dementia (CDR = 1.0)

MCI (CDR = 0.5), more specifically a PPA variant, namely …

2. What is the most likely primary etiology? (choose one)

Alzheimer

Vascular

FTLD spectrum

Lewy body disease

Tauopathy (CBD, PSP)

Other neurodegenerative disorder, namely

No neurodegenerative disorder, namely

Unclear / deferred

3. Remark (e.g., suspicion of underlying pathology within FTLD spectrum / CBS / suspicion of mutation / specify Lewy body disease)

4. Is there mixed pathology?

No

Yes, with….

5. Etiological differential diagnosis? (multiple possible)

Alzheimer

Vascular

FTLD spectrum

Lewy body disease

Tauopathy (CBD, PSP)

Other neurodegenerative disorder, namely

No neurodegenerative disorder, namely

Unclear / deferred

6. Degree of certainty about underlying etiology?

Percentage (0-85%):

7. Reason for low certainty? (multiple possible)

Suspected mixed pathology

Atypical clinical presentation, if so, describe the clinical presentation:

Conflicting/inconclusive information from other tests such as MRI and CSF or clinic and MRI, if so, which results conflict:

8. What is the follow-up plan?

8.1 Additional investigation (besides tau PET scan): (multiple possible)

None

CSF

FDG PET

Amyloid PET

DaT SPECT

2nd clinical assessment

Speech assessment

Referral to psychiatry

Other, namely…

8.2 Pharmacological intervention: (multiple possible)

None

Consider cholinesterase inhibitor

Other pharmacological therapy, namely…

8.3 Care: (multiple possible)

None

Care meeting

Indication for procedure at CBR

Case manager

Day care

Nursing home

Paramedics

8.4 Follow-up: (choose one)

After additional investigation

(Semi-)annual check-up

Referral

Discharge

9. Would you prescribe disease-modifying therapy for Alzheimer's disease based on the current data if it were available?

Yes

No

AFTER TAU-PET

Date of Completion:

Date of Disclosure Appointment:

Treating physician:

1. What is the syndrome diagnosis? (choose one)

MCI (CDR = 0.5)

Dementia (CDR = 1)

MCI (CDR = 0.5), more specifically PPA variant, namely …

Neurology, other, namely

Psychiatry

Deferred

2. What is the most probable primary etiology? (choose one)

Alzheimer

Vascular

FTLD spectrum

Lewy body disease

Tauopathy (CBD, PSP)

Other neurodegenerative disorder, namely

No neurodegenerative disorder, namely

Unclear / deferred

3. Is there mixed pathology? (choose one)

No

Yes, namely …

4. Etiological differential diagnosis? (multiple possible)

Alzheimer

Vascular

FTLD spectrum

Lewy body disease

Tauopathy (CBD, PSP)

Other neurodegenerative disorder, namely

No neurodegenerative disorder, namely

Unclear / deferred

5. What is your degree of certainty about the underlying etiology now?

…… %

6. Does the follow-up plan change?

6.1 Additional investigation: (multiple possible)

Unchanged

Changed, add…

Changed, no longer necessary…

6.2 Pharmacological therapy (multiple possible)

Unchanged

Changed, consider a cholinesterase inhibitor

Changed, no longer consider cholinesterase inhibitor

Other change, namely…

6.3 Care

Unchanged

Changed, namely…

6.4 Follow-up (multiple possible)

Unchanged

Changed, namely…

After additional investigation

(Semi-)annual check-up

Referral

Discharge

7. Would you prescribe disease-modifying therapy for Alzheimer's disease based on the current data if it were available?

Yes

No

1 YEAR AFTER TAU-PET

Date of Completion:

Date of Follow-Up Appointment:

Treating hysician:

1. What is the syndrome diagnosis? (choose one)

MCI (CDR = 0.5)

Dementia (CDR = 1-3)

MCI (CDR = 0.5), more specifically PPA variant, namely …

Neurology, other, namely

Psychiatry

Deferred

2. What is the most probable primary etiology? (choose one)

Alzheimer

Vascular

FTLD spectrum

Lewy body disease

Tauopathy (CBD, PSP)

Other neurodegenerative disorder, namely

No neurodegenerative disorder, namely

Unclear / deferred

3. Is there mixed pathology? (choose one)

No

Yes, namely…

4. Etiological differential diagnosis? (multiple possible)

Alzheimer

Vascular

FTLD spectrum

Lewy body disease

Tauopathy (CBD, PSP)

Other neurodegenerative disorder, namely

No neurodegenerative disorder, namely

Unclear / deferred

5. If the diagnosis has changed compared to the diagnosis just after the tau-PET scan, indicate on what basis this change is (e.g., additional investigation, clinical course, …)

6. What is your degree of certainty about the underlying etiology now?

- …… %

7. Does the follow-up plan change?

7.1 Additional investigation: (multiple possible)

Unchanged

Changed, add…

Changed, no longer necessary…

7.2 Pharmacological therapy (multiple possible)

Unchanged

Changed, consider a cholinesterase inhibitor

Changed, no longer consider cholinesterase inhibitor

Other change, namely…

7.3 Care

Unchanged

Changed, namely…

After additional investigation

(Semi-)annual check-up

Referral

Discharge

8. Would you prescribe disease-modifying therapy for Alzheimer's disease based on the current data if it were available?

Yes

No
